# Massive Ovarian Edema Presenting With Abnormal Uterine Bleeding: A Case Report

**DOI:** 10.7759/cureus.102691

**Published:** 2026-01-31

**Authors:** Harneet S Randhawa, Aradhana A Shanmughan

**Affiliations:** 1 Department of Radiodiagnosis, Aarthi Scans and Labs, Pune, IND; 2 Department of Radiology, Sassoon General Hospital, Pune, IND; 3 Department of Radiology, Government Medical College, Baramati, Baramati, IND; 4 Department of Radiology, Sree Balaji Medical College and Hospital, Chennai, IND

**Keywords:** abnormal uterine bleeding, adnexal torsion, massive ovarian edema, ovarian lesions, ovarian torsion

## Abstract

Massive ovarian edema (MOE) is a rare, tumor-like, benign condition characterized by diffuse ovarian enlargement. It is mostly associated with partial/intermittent adnexal torsion, resulting in impaired venous and lymphatic drainage of the ovaries. Owing to its nonspecific clinical presentation and tumor-like imaging appearance, MOE is often misdiagnosed as an ovarian neoplasm, leading to unnecessary oophorectomies. We report a case of MOE in a young nulligravid woman who presented with abnormal uterine bleeding. Following an initial inconclusive clinical evaluation and ultrasonography, the diagnosis was eventually confirmed by magnetic resonance imaging (MRI) of the pelvis, and appropriate treatment could be undertaken. Due to the rarity of incidence and atypical presentation, we would like to add this case to the existing scientific literature.

## Introduction

Massive ovarian edema (MOE) was first described and published by Kalstone et al. in 1969 [1,2]. MOE is a rare, pseudo-tumorous entity characterized by diffuse enlargement of the ovary due to the accumulation of interstitial fluid within the ovarian stroma. It predominantly affects adolescents and young women [2]. It closely mimics ovarian neoplasms in both clinical presentation and imaging appearance, often leading to a diagnostic dilemma [3].

The underlying pathophysiological mechanism in the development of MOE involves recurrent partial torsion of the meso-ovarium, leading to impaired venous and lymphatic drainage while preserving arterial inflow. This process results in progressive stromal edema without necrosis. Focal stromal luteinization and production of androgens may occur [3].

This pathology is mostly unilateral, with the right ovary affected more commonly than the left. Clinically, patients often present with intermittent pelvic pain or a palpable pelvic mass lesion. Rarely, they may present with polycystic ovarian syndrome-like features such as menstrual irregularities, infertility, hirsutism, and virilization due to stromal luteinization. Ascites and Meigs’ syndrome may also occur, further complicating the diagnosis [2].

Imaging plays a crucial role in diagnosis. Ultrasonography is often non-specific and shows a complex solid-cystic mass or an enlarged hypoechoic ovary with peripherally displaced follicles. Magnetic resonance imaging (MRI) is the imaging investigation of choice for MOE and ovarian lesions. In MOE, the enlarged ovary shows a homogeneously low signal intensity on T1-weighted images and a high signal intensity with peripherally located follicles on T2-weighted images. Contrast enhancement can often be elicited in the enlarged ovary. Diffusion restriction is not a feature of MOE. Computed tomography is not generally advocated for ovarian pathologies [4,5].

Accurate and timely diagnosis allows conservative surgical management with preservation of ovarian function [6]. We present a rare case of MOE presenting with abnormal uterine bleeding, underscoring diagnostic challenges and management considerations.

## Case presentation

About a year ago, a 24-year-old nulligravid woman presented to her gynecologist with complaints of abnormal uterine bleeding and mild intermittent pelvic pain. She had been experiencing these symptoms for 10 months, but she had ignored them, as her mother also experienced abnormal uterine bleeding. There was no history of fever, weight loss, bowel or urinary symptoms, or any prior surgery. On examination, the patient was hemodynamically stable. No abdominal or pelvic tenderness could be elicited. The patient was advised to follow up within a week with tumor markers and an ultrasonography report.

The results of her serum tumor markers were within normal limits (Table 1).

**Table 1:** Tumor markers Alpha-Fetoprotein (AFP), Beta-Human Chorionic Gonadotrophin (β-hCG), Lactate Dehydrogenase (LDH), Carcinoembryonic Antigen (CEA), Cancer Antigen 125 (CA-125) [7-10]. Nanogram (ng), Milliliter (mL), Liter (L), Milli International Units (mIU), and Units (U).

Tumor Markers	Results	Reference Range
AFP	3 ng/mL	< 10 ng/mL
β-hCG	1 mIU/mL	< 5 mIU/mL
LDH	152 U/L	140–280 U/L
CA-125	2 U/mL	< 35 U/mL
CEA	0 ng/mL	< 3.4–5 ng/mL

Transvaginal ultrasonography revealed a heterogeneously hypoechoic, large left ovarian lesion with a few peripheral cysts/follicles and internal vascularity. The findings were inconclusive, and further testing was suggested to rule out neoplastic pathology. Hence, the patient came to our department for a MRI scan of the pelvis two weeks later.

**Figure 1:**
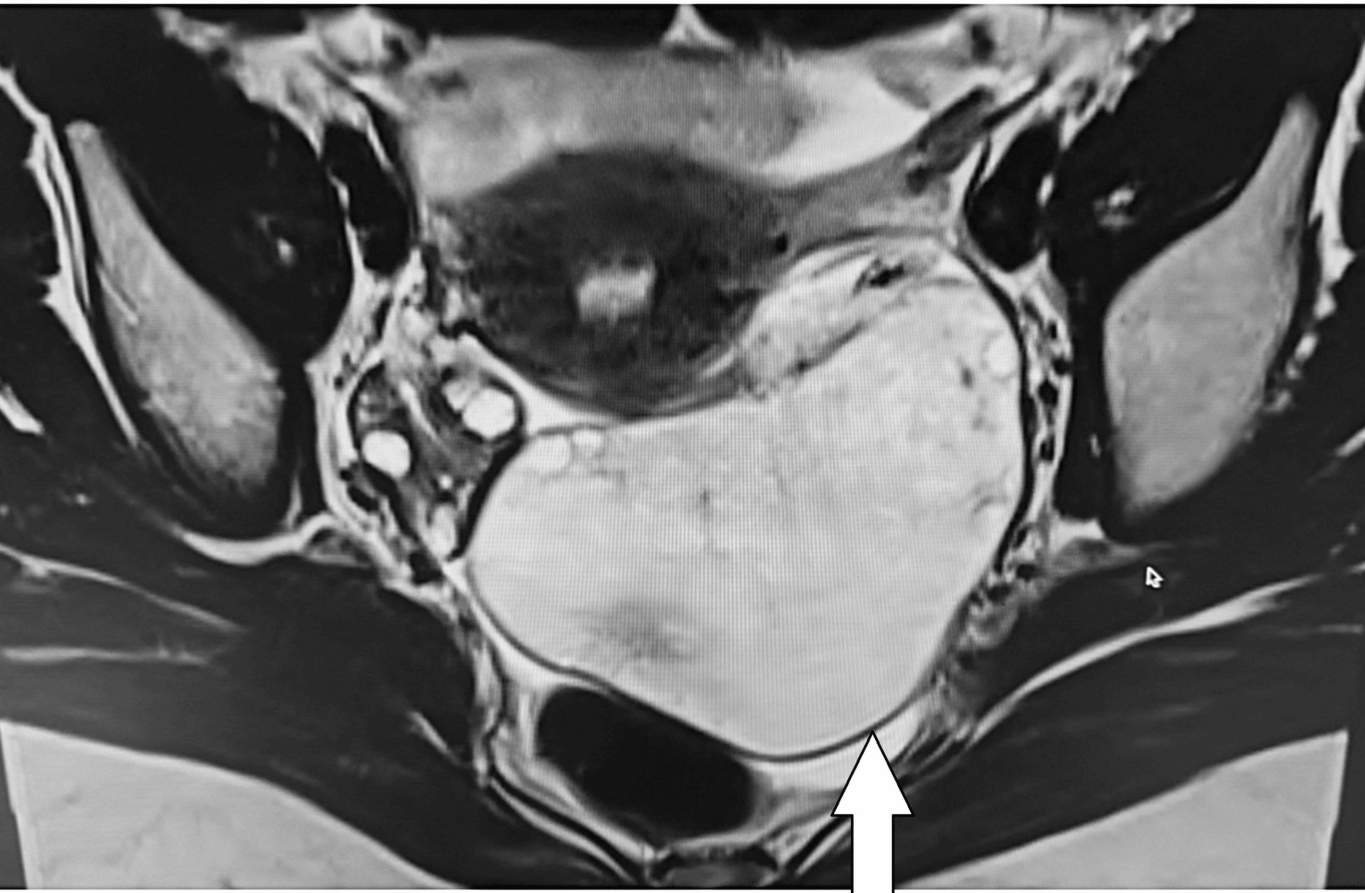
Axial T2-weighted pelvic MRI image showing homogeneously high signal intensity within the enlarged left ovary (white arrow).

**Figure 2:**
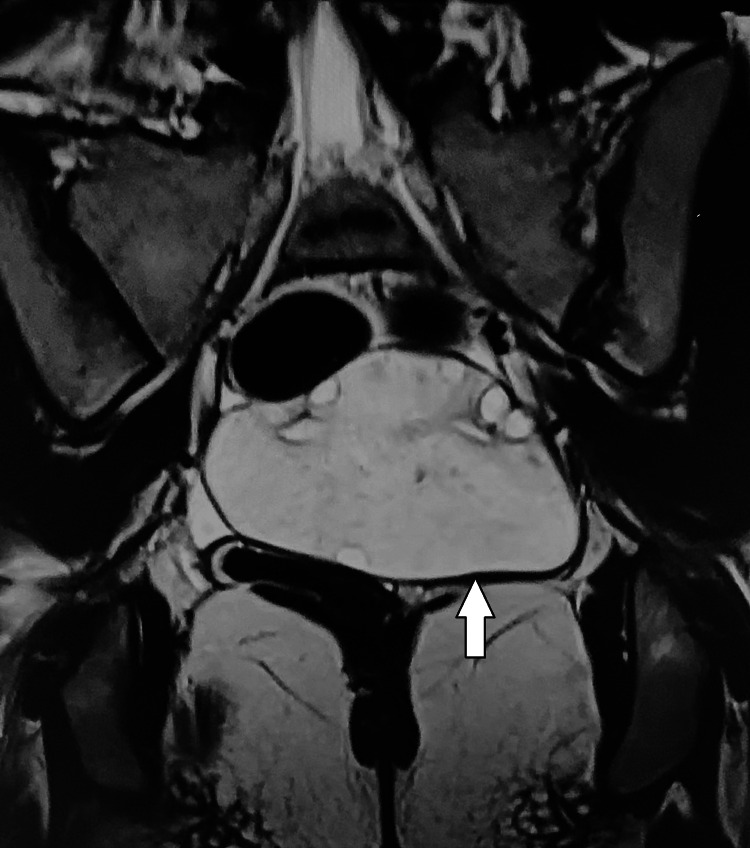
Coronal T2-weighted pelvic MRI image showing homogeneously high signal intensity within the enlarged left ovary (white arrow).

**Figure 3:**
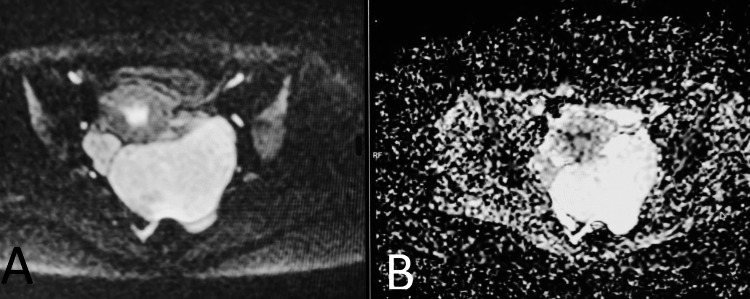
(A) Diffusion-weighted imaging (DWI) and (B) apparent diffusion coefficient (ADC). No diffusion restriction is seen in the lesion.

After confirming the clinical history and obtaining detailed informed consent, a pelvic MRI was performed, which demonstrated an enlarged left ovary extending into the pouch of Douglas, measuring approximately 9.4 × 6.3 × 5.3 cm. It showed diffuse T2 hyperintensity and a few follicles along the periphery. There was no evidence of diffusion restriction, hemorrhage, or calcification in the affected ovary. The left ovarian pedicle appeared mildly bulky. These imaging findings favored a diagnosis of MOE over ovarian neoplasm and polycystic ovarian syndrome (Figures 1-3).

Laparoscopic surgery was conducted by her gynecologist in the following week, which revealed a markedly enlarged left ovary with partial torsion. Detorsion and ovarian fixation were done. A wedge biopsy was obtained, which showed stromal edema and ruled out neoplastic etiology. The postoperative course was uneventful. Follow-up ultrasonography conducted six months after surgery revealed a reduction in the size of the left ovary. The patient also reported regular menstrual cycles eight months after the surgery.

## Discussion

MOE is an uncommon pseudotumor of the ovary. It has an atypical clinical presentation and often presents as a diagnostic dilemma, mimicking a neoplasm [6].

The underlying pathophysiological mechanism in the development of MOE involves recurrent partial torsion of the meso-ovarium [3]. Our patient also had a history of intermittent lower abdominal pain, which can be retrospectively attributed to repeated partial torsions, consistent with the pathogenesis.

The pathology is mostly unilateral, with the right ovary affected more commonly than the left [2]. Our patient also had unilateral involvement, but it affected the left ovary, which is relatively uncommon.

Patients generally present with intermittent pelvic pain or a palpable pelvic mass lesion. Rarely, they may present as polycystic ovarian syndrome-like features and virilization due to stromal luteinization, which can further delay the diagnosis [2]. Our patient had a primary complaint of abnormal uterine bleeding, which can be attributed to luteinization of ovarian stroma.

Ultrasonography is often non-specific and shows a complex solid-cystic mass or an enlarged hypoechoic ovary with peripherally displaced follicles. Internal vascularity is often appreciated on Doppler. Thus, ultrasonography cannot rule out neoplastic pathology [4]. Our patient also had inconclusive findings on the ultrasound scan and required further evaluation to rule out an ovarian neoplasm.

MRI is the imaging modality of choice for MOE [4]. The pelvic MRI of our patient demonstrated an enlarged left ovary with diffuse T2 hyperintensity and a few follicles along the periphery. These findings favored MOE. The lack of diffusion restriction made the possibility of a neoplasm unlikely. The left ovarian pedicle appeared mildly bulky, adding more imaging evidence in favor of MOE.

MOE is often misdiagnosed, and thus, most patients have been subjected to oophorectomies in the past. The management of MOE in young women should prioritize preservation of the ovaries [6]. Conservative surgery with a histopathology correlation showing stromal edema and no neoplastic component helped preserve the ovary in our patient. Follow-up ultrasonography showed a reduction in the size of the ovary and resolution of the patient's symptoms. This case underscores the importance of a timely and accurate diagnosis of MOE with MRI.

## Conclusions

MOE is an uncommon but important mimic of ovarian neoplasms. Early recognition and accurate diagnosis with MRI helps avoid unnecessary oophorectomies. Conservative surgery with intraoperative wedge resection to confirm the diagnosis is often enough to resolve patients' symptoms and preserve fertility. Further longitudinal follow-up studies with larger sample sizes are required to assess the long-term benefits of conservative surgeries.
